# Light-responsive metal–organic framework sheets constructed smart membranes with tunable transport channels for efficient gas separation

**DOI:** 10.1039/d1ra06814h

**Published:** 2021-12-22

**Authors:** Qingping Xin, Meixue Zhao, Jianping Guo, Dandan Huang, Yinan Zeng, Yuhang Zhao, Teng Zhang, Lei Zhang, Shaofei Wang, Yuzhong Zhang

**Affiliations:** State Key Laboratory of Separation Membranes and Membrane Processes, School of Materials Science and Engineering, Tiangong University Tianjin 300387 P. R. China xinqingping@tiangong.edu.cn zhangyz2004cn@vip.163.com; College of Chemistry and Chemical Engineering, Hunan University Changsha 410082 China

## Abstract

Exploring a new type of smart membrane with tunable separation performance is a promising area of research. In this study, new light-responsive metal–organic framework [Co(azpy)] sheets were prepared by a facile microwave method for the first time, and were then incorporated into a polymer matrix to fabricate smart mixed matrix membranes (MMMs) applied for flue gas desulfurization and decarburization. The smart MMMs exhibited significantly elevated SO_2_(CO_2_)/N_2_ selectivity by 184(166)% in comparison with an unfilled polymer membrane. The light-responsive characteristic of the smart MMMs was investigated, and the permeability and selectivity of the Co(azpy) sheets-loaded smart MMMs were able to respond to external light stimuli. In particular, the selectivity of the smart MMM at the Co(azpy) content of 20% for the SO_2_/N_2_ system could be switched between 341 and 211 *in situ* irradiated with Vis and UV light, while the SO_2_ permeability switched between 58 Barrer and 36 Barrer, respectively. This switching influence was mainly ascribed to the increased SO_2_ adsorption capacity in the visible light condition, as verified by adsorption test. The CO_2_ permeability and CO_2_/N_2_ selectivity of MMMs in the humidified state could achieve 248 Barrer and 103.2, surpassing the Robeson's upper bound reported in 2019.

## Abstract 2

New light-responsive MOF sheets were synthesized to fabricate a smart membrane, which displays switchable gas separation performance stimulated with UV-Vis light.

## Graphical abstract

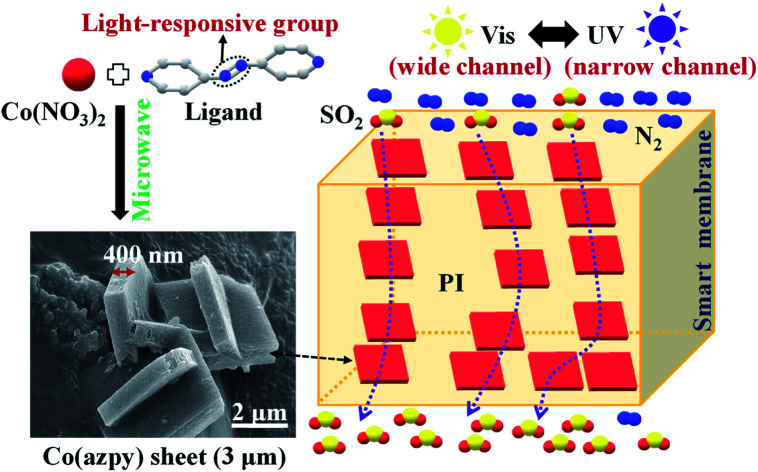

## 1. Introduction

With the development of economy, energy consumption increases, and thus leads to new environmental burdens and hazards for human health.^1–4^ Hence, the exploration of new ways to separate air pollutants such as CO_2_ and SO_2_ is a challenging scientific and technical issue.^5–7^ The emissions of CO_2_ and SO_2_ are mainly from the combustion of coal caused by industrial processes and heavy oil used by vehicles, respectively.^8–10^ Membrane technology as one method for gas separation is promising, and has attracted the attention of researchers.^11–14^ Mixed matrix membranes (MMMs) prepared by incorporating the filler phase into the polymer phase have been deemed as potential membrane materials for overcoming the trade-off between gas permeability and selectivity.^15,16^ The integration of the merits for the filler phase and polymer matrix is deemed to be a promising way to surpass the trade-off limit, achieving enhanced permeability and selectivity simultaneously.^17–19^ The rational design of advanced two-dimensional (2D) fillers was conducted to reach predominant permeability and selectivity of MMM.^20–22^ The 2D fillers, including graphene oxide,^20,23–26^ metal–organic framework (MOF) sheets,^22,27^ and MXene sheets,^28–31^ were introduced into the polymer to fabricate MMMs. Among them, MOF sheets have attracted the attention of researchers due to the merits of structural characteristics, which consist of ultrathin thickness in nanoscale, porous channels in micropores, and rich interaction sites. MOF sheets are promising and alternative candidate fillers for preparing MMMs to further elevate the gas separation performance.

Recently, several CO_2_ separation and MOF gas separation methods were investigated.^32,33^ Peng *et al.* prepared a light-responsive metal–organic framework hybrid membrane with high on/off photo switchable proton conductivity.^34^ They also investigated gas transport through two-dimensional nanoslits, and the construction of 2D nanoslits and mechanisms for gas transport were summarized.^35^ Helms *et al.* prepared diamine-appended Mg_2_(dobpdc) nanorods as phase-change fillers in mixed matrix membranes for efficient CO_2_/N_2_ separations.^36^ Chen *et al.* explored porous metal–organic frameworks for gas separation and purification, and concluded that several approaches were developed to systematically tune the pores and to immobilize the functional sites.^37^

A majority of research studies of sheet-doped MOFs MMMs have been conducted for gas separation.^29^ Yang *et al.* prepared CuBDC nanosheets-doped MMMs, and the CO_2_/CH_4_ selectivity of MMMs significantly improved.^38^ Rodenas *et al.* explored MMMs loaded with CuBDC nanosheets with pore diameters of ∼0.52 nm, which also significantly increased the CO_2_ permeability.^39^ This result concluded that MOF sheets were beneficial for enhancing the CO_2_ permeability, exhibiting promising candidates to surpass the trade-off effect of membranes. A novel oriented and penetrating ZIF-7@PI mixed matrix membrane (MMM) with ZIF-7 loading 50 wt% was developed by Huang,^40^ and the ZIF-7@PI MMM displayed high selectivity for H_2_/CO_2_ and H_2_/CH_4_ systems with 91.5 and 128.4, respectively.

Apart from the MMM itself, the gas separation performance can be further improved by adopting an external stimulus.^41^ Recently, external stimuli, such as temperature,^42^ light, and electric field,^43^ have been used to elevate the gas separation performances. Among these external stimuli, light is thought to be the most promising one because of its ease of control, fast-responsiveness, and non-contact characteristics. Generally, light-responsive fillers have been introduced into polymers to tune the gas permeability through structure transformation simulated by light. Peng *et al.* prepared Zr-Fc MOF nanosheets, which exhibited the potential for gas separation.^44^ The MOFs (Zr-Fc MOF)-based nanosheets are synthesized as porous supports to fabricate a Zr-Fc MOF-supported ionic liquid membrane (Zr-Fc-SILM) for highly efficient CO_2_ separation. The micropores of the Zr-Fc MOF nanosheets not only provide extra paths for CO_2_ transport with CO_2_ permeability up to 145.15 GPU, but also endow the Zr-Fc-SILM with high selectivity (216.9) of CO_2_/N_2_. Ladewig *et al.* prepared light-responsive MOF (Azo-UiO-66) for post combustion CO_2_ capture.^40^ Azo-UiO-66 was incorporated into the polymer to form mixed matrix membranes (MMMs), and the results showed significantly increased CO_2_ permeability and CO_2_/N_2_ selectivity. Huang *et al.* developed a new kind of light-induced smart MOF membrane, and the selectivity of the H_2_/CO_2_ system can be switched reversibly between 21.3 and 43.7 under *in situ* irradiation with UV and Vis light, respectively.^45^ This switching effect is mainly caused by regulated CO_2_ adsorption sites in the UV-Vis state, as proven by independent adsorption studies.

Herein, light-responsive MOF [Co(azpy)] sheets were synthesized for the fabrication of a light-responsive membrane for efficient gas separation. The light-responsive sheets are 2D porous materials with a rich pore structure, which are considered to offer extra pathways for gas transport, and thus elevate gas permeability. Meanwhile, the microporous channels of Co(azpy) endow the MMMs with high CO_2_(SO_2_)/N_2_ selectivity by tuning the pore size. As a consequence, the Co(azpy) shows excellent gas separation performance, which solves the trade-off effect. In addition, the Co(azpy) is a photo agent capable of converting the structure under visible light and ultraviolet light illumination, which is also being investigated.

## 2. Experimental

### 2.1 Chemicals and materials

**Scheme 1:**
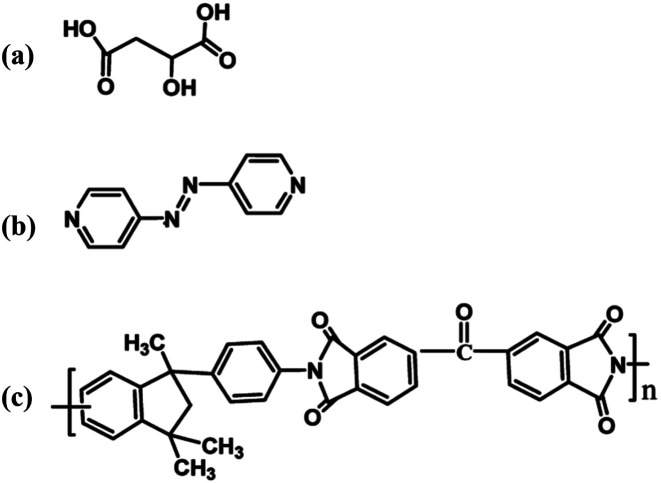
Chemical structure of (a) l-malic acid, (b) 4,4′-bisazobipyridine (azpy), and (c) Matrimid® 5218 (PI).

Co(NO_3_)_3_·6H_2_O and l-malic acid (Scheme 1a) were purchased from Aladdin Chemical Co., Ltd. 4,4′-Bisazobipyridine (azpy, Scheme 1b) was obtained from Sigma Aldrich Chemical Co. *N*,*N*-Dimethylformamide (DMF) and *N*,*N*-dimethylacetamide were obtained from Kemiou Chemical Co., Ltd. (Tianjin, China). Matrimid® 5218 (PI, Scheme 1c) was obtained from Alfa Aesar China Co., Ltd. SO_2_, CO_2_ and N_2_ were purchased from Tianjin Huanyu Co., Ltd.

### 2.2 Preparation process of light-responsive MOF [Co(azpy)] sheets

**Scheme 2:**
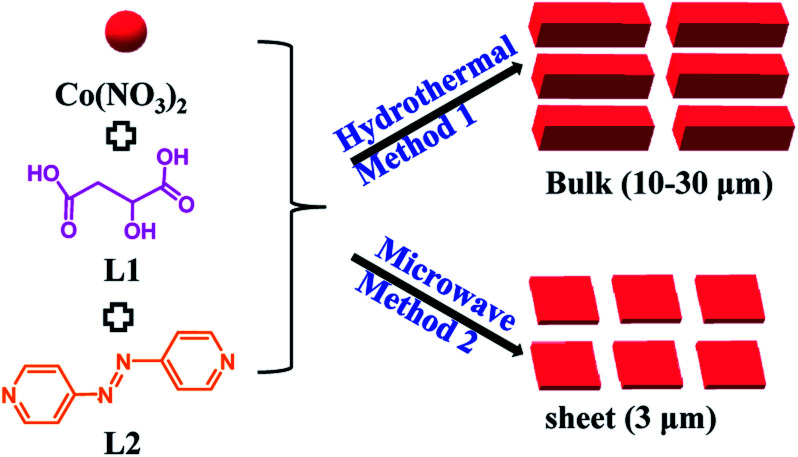
Synthesis process of the light-responsive MOF [Co(azpy)].

The light-responsive MOF [Co(azpy)] was prepared by two methods, as follows (Scheme 2). Taking the microwave method for an example, Co(NO_3_)_3_·6H_2_O (0.58 g) and 4,4′-bisazobipyridine (0.18 g) were separately dispersed in 16 ml DMF and 8 ml DMF, respectively. l-Malic acid (0.268 g) was dissolved in water (1 ml), and then DMF (8 ml) was added. The abovementioned solutions were mixed with stirring for 30 min, and were then transferred to a microwave oven to heat at 120 °C for 60 min. The as-prepared MOF [Co(azpy)] was centrifuged and further washed, obtaining the sheets products (∼3 μm). The preparation process of the hydrothermal method was almost similar, but the mixed solution was placed in a muffle furnace at 120 °C for 8 h, obtaining the bulk products (10–30 μm).

### 2.3 Fabrication of membranes

Four copies of PI (0.6 g) were dissolved in DMAc to get a 5 wt% PI solution, respectively. Co(azpy) fillers with predetermined amounts (0.06 g, 0.09 g, and 0.12 g) were separately sonicated in DMAc for 6 h to obtain the filler suspension. Subsequently, the Co(azpy) suspension was added into the PI-DMAc solution to form the MMMs casting solution. The casting solution was poured into a glass plate after stirring for 12 h, and then was processed at 50 °C and 80 °C for 12 h and 12 h in succession. The as-prepared membranes were peeled from the glass plate, and placed in the vacuum oven at 120 °C. The unfilled PI membrane was also prepared using the same method. The as-prepared MMMs loaded with Co(azpy) were referred to as PI/Co(azpy)-*X*, where *X* (*X* = 10, 15, 20) represented the weight percentage of Co(azpy) relative to the PI polymer. All membrane thicknesses were in the range of 30–70 μm.

### 2.4 Characterization

The morphologies of Co(azpy) and MMMs were observed by field emission scanning electron microscope (SEM, S-4800). The crystalline property and chemical structure of Co(azpy) and MMMs were measured by X-ray diffraction (XRD, D8 Advance) and Fourier transform infrared spectroscopy (FT-IR, BRUKER Vertex 70), respectively. The thermal stability of Co(azpy) and MMMs was explored by thermal gravimetrical analysis (TGA, STA409PC). N_2_ adsorption–desorption and CO_2_ adsorption isotherm measurements of Co(azpy) were tested at 298 K (ASAP 2020). Differential scanning calorimetry (DSC, D8-DISCOVER) was conducted to measure the glass transition temperatures (*T*_g_) of the membranes. The SO_2_ adsorption capacity of Co(azpy) was measured by gravimetric method under visible light and ultraviolet light, respectively. The light-responsive characteristic was investigated by TU-1901 ultraviolet-visible absorption spectrometry.

### 2.5 Gas permeation tests of Co(azpy)-loaded MMMs

**Scheme 3:**
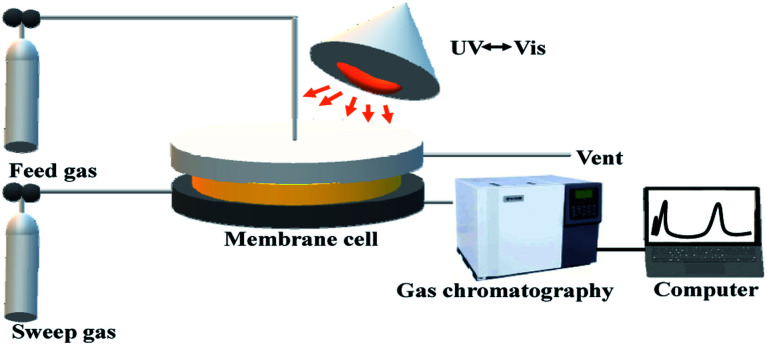
The diagrammatic sketch of the gas membrane separation apparatus.

Gas separation performances of the Co(azpy)-loaded membranes were measured by a constant pressure/variable volume method, as reported. The CO_2_, SO_2_ and N_2_ permeabilities were conducted. The gas membrane separation apparatus is shown in Scheme 3, which displayed the gas separation process. The CO_2_/N_2_ pure gas system and SO_2_/N_2_ (1 : 9 in vol) mixed gas were tested as feeding gases. The permeability (*P*_i_, Barrer, and 1 Barrer = 10^−10^ cm^3^ (STP) cm (cm^−2^ s^−1^ cmHg^−1^) could be calculated by eqn (1),1
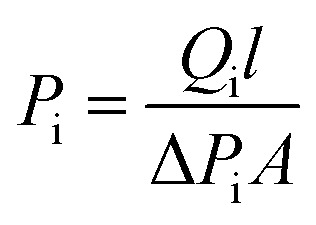


The selectivity of the CO_2_(SO_2_)/N_2_ (*α*_ij_) system was calculated by eqn (2),2
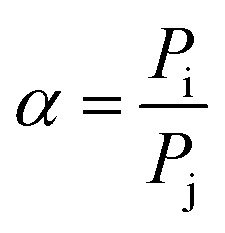


## 3. Results and discussion

### 3.1 Characterization of the nanosheets

**Fig. 1:**
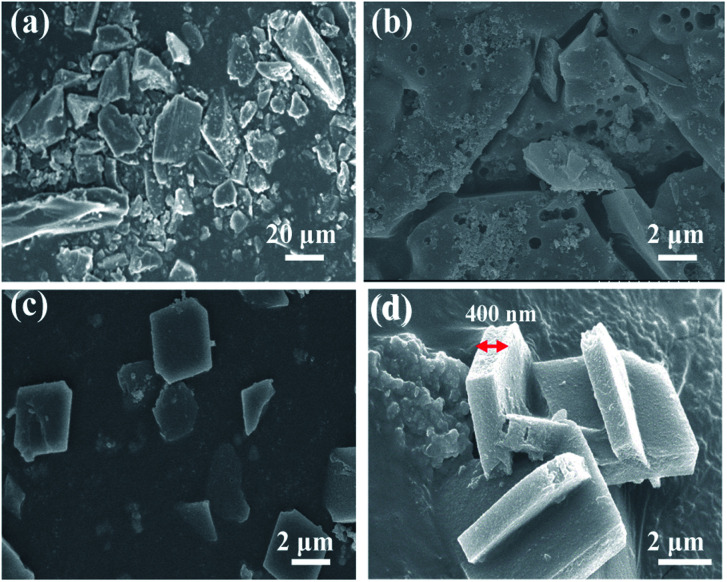
SEM images of Co(azpy) prepared by (a, b) hydrothermal method and (c, d) microwave method, respectively.

The morphology of Co(azpy) was characterized by scanning electron microscopy, as shown in Fig. 1. The particle size of Co(azpy) synthesized by hydrothermal reaction method is large (about 10–30 μm) and is not uniform. However, Co(azpy) particles synthesized by microwave method have regular morphology and uniform size, and the particle size of about 3 μm is much smaller than those of the Co(azpy) particles synthesized by hydrothermal method. From Fig. 1(c), we could obtain the sheet-shaped morphology of Co(azpy) and the particle size. From Fig. 1(d), we could obtain the thickness of the Co(azpy) sheet at about 400 nm. The Co(azpy) synthesized by microwave method with small particle size may possess a large specific surface area, which is beneficial to adsorbing SO_2_.

**Fig. 2:**
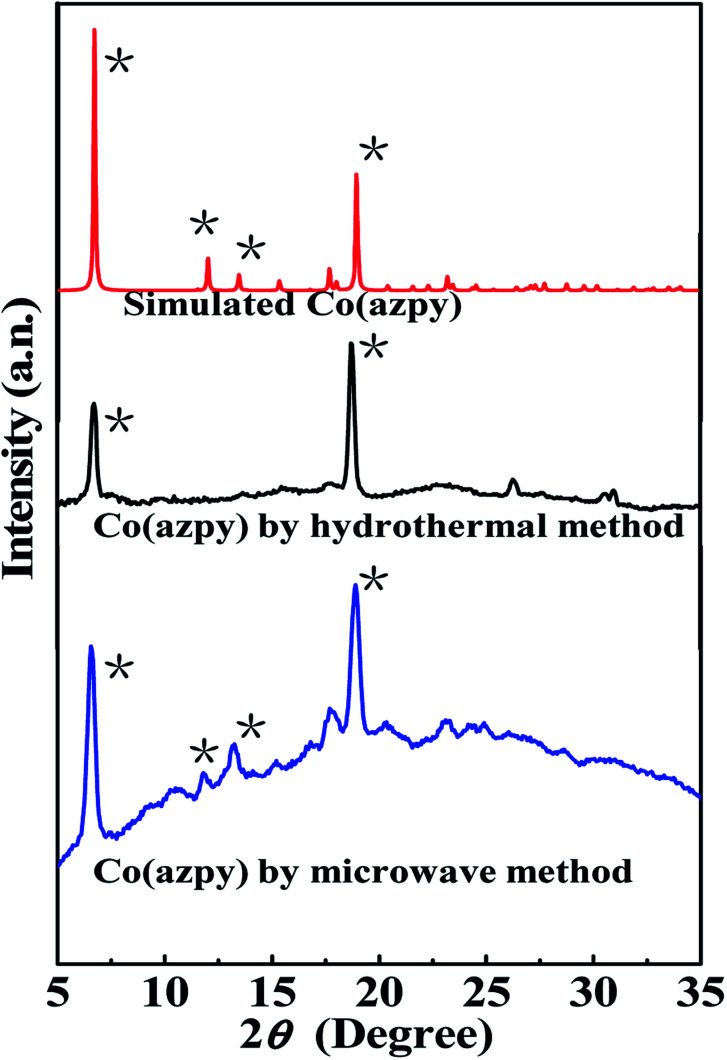
XRD patterns of Co(azpy) prepared by both hydrothermal and microwave methods.

As shown in Fig. 2, the XRD patterns of the hydrothermal and microwave synthesis methods of Co(azpy) exhibit basically the same peak positions. The peak positions of Co(azpy) prepared by the two methods are identical to those of the simulated Co(azpy), indicating that the crystal structure of Co(azpy) synthesized by these two methods has no significant variation in comparison with the simulated Co(azpy). The peak position is identical to the data, as reported in the literature,^46^ which indicates that Co(azpy) was successfully synthesized by both hydrothermal and microwave reaction methods.

**Fig. 3:**
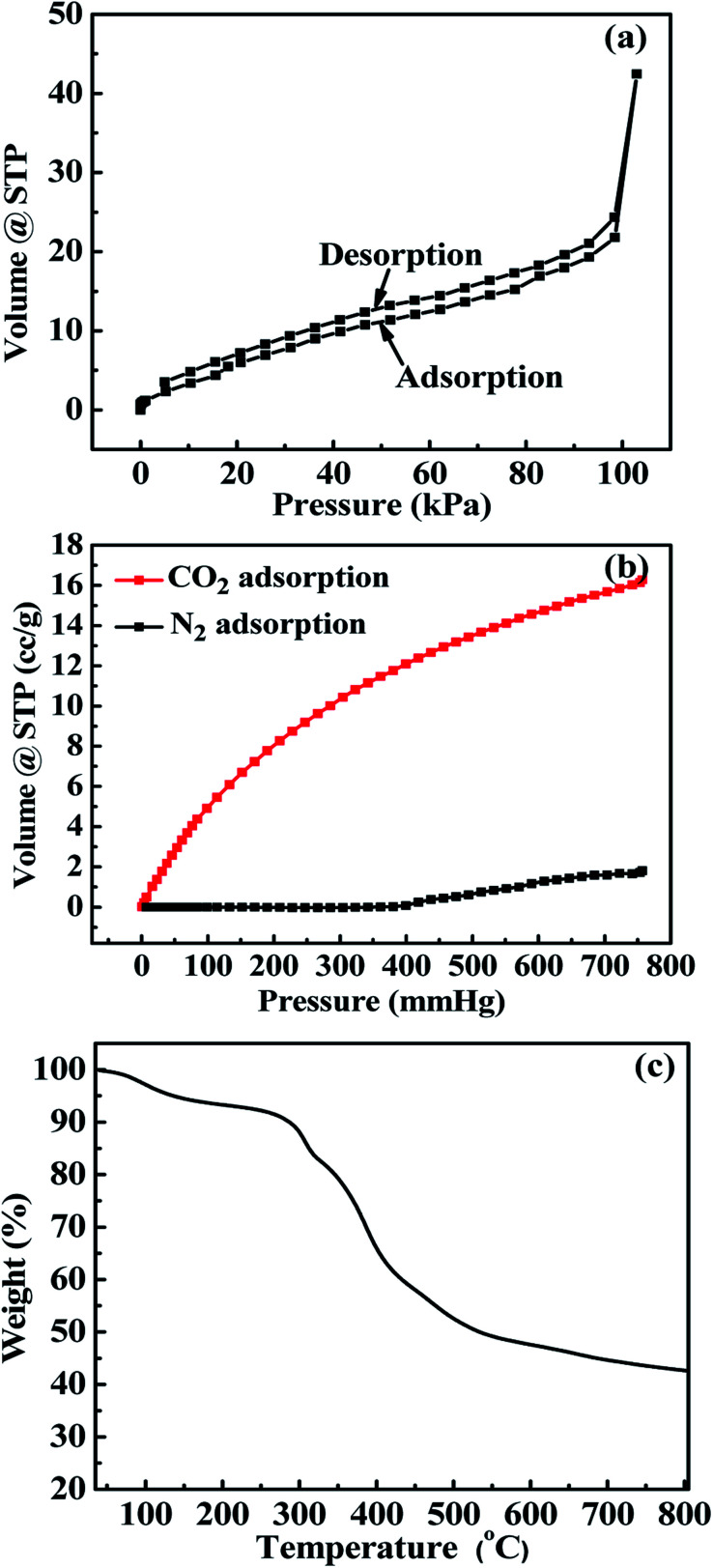
(a) N_2_ adsorption–desorption isotherms; (b) CO_2_ and N_2_ adsorption capacities; and (c) TGA plot of Co(azpy) prepared by microwave method.

The sheet-shaped Co(AzDC) of about 3 μm prepared by microwave method displays relatively uniform and small particle size, which was chosen for further investigation. The N_2_ adsorption–desorption isotherm of Co(azpy) at 77 K is shown in Fig. 3(a). Co(azpy) exhibits the microporous structure, which is in accordance with the result (pore size ∼7.0 × 6.2 Å^2^), as reported in the literature.^46^ The adsorption isotherm of Co(azpy) for N_2_ and CO_2_ at 273 K is displayed in Fig. 3(b). It can be seen that the adsorption selectivity of CO_2_/N_2_ is high at low pressure below 400 mmHg pressure, and the adsorption selectivity descends as the pressure ascends. However, the Co(azpy) retains high adsorption selectivity of about 10 at 760 mmHg pressure. In order to investigate the thermal stability of Co(azpy) synthesized by microwave method, a TGA test was conducted. As shown in Fig. 3(c), the degradation process of Co(azpy) is mainly divided into two stages. The first stage is the release of solvent molecules in the range of 50–150 °C, and the second stage is the degradation of azpy in the range of 270–500 °C. The relatively high stable temperature of Co(azpy) is up to 270 °C.

**Fig. 4:**
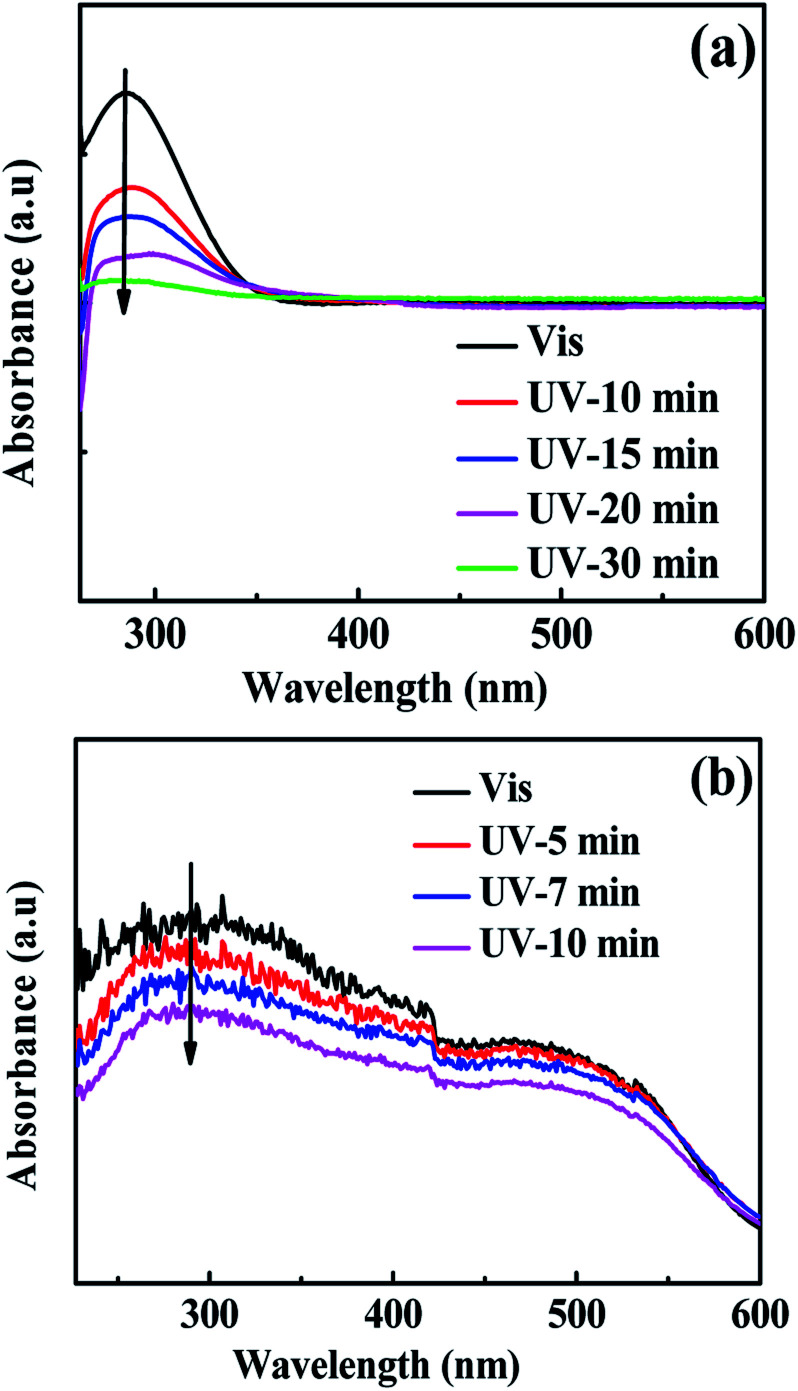
The ultraviolet-visible (UV-Vis) spectrum of (a) the azobenzene-4,4-dicarboxylic acid ligand (azpy) solution and (b) Co(azpy); the infrared spectra of the (c) Co(azpy) particles.

Fig. 4 displays the ultraviolet-visible spectra of the ligand (azpy) and light-responsive MOFs [Co(azpy)]. From Fig. 4(a), it can be seen that the ligand azpy displays a strong absorption peak at 290 nm, which corresponded to the absorption peak of the π–π* transition of the anti-formula structure.^47^ Obviously, azpy exhibits a certain red shift after ultraviolet stimulation. This may be attributed to the nitrogen atom on the pyridine group, which possesses a lone pair of electrons and participates in the conjugation of the benzene ring, influenced by the ultraviolet light to the transition state. Under ultraviolet light, the intensity of the maximum absorption peak of the ligand azpy at 290 nm decreases, and the absorbance of the n–π* transition at 460 nm is almost not changed. Obviously, the decrease in the intensity of the maximum absorption peak of the ligand azpy is caused by the structural transformation of azpy from *trans* to *cis*. Fig. 4(b) displays the UV-Vis spectrum of the Co(azpy) powder. It can be seen that Co(azpy) exhibits strong broad peaks around 295 nm and 480 nm, respectively, and the intensity of the absorption peak at 295 nm descends as the time of the ultraviolet light stimulation increases. This indicates that in Co(azpy), the ligand azpy already has undergone structural transformation from *trans* to *cis*.

**Fig. 5:**
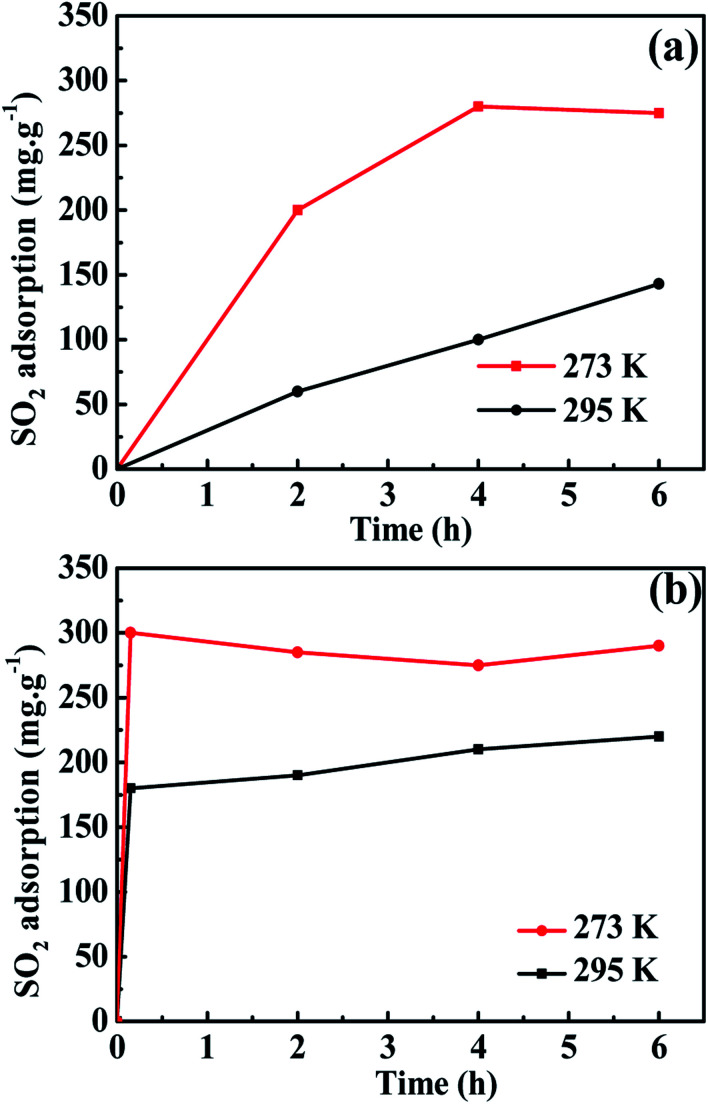
The SO_2_ adsorption capacity of Co(azpy) prepared by (a) hydrothermal method and (b) microwave method under different temperatures.

The adsorption processes of SO_2_ for the light-responsive MOF [Co(azpy)] synthesized by hydrothermal method tested at 295 K and 273 K, respectively, are displayed in Fig. 5(a). At 273 K, the Co(azpy) dynamic adsorption reaches equilibrium at 4 h, and the adsorption capacity is 285 mg g^−1^. At 295 K, the maximum adsorption capacity of Co(azpy) to SO_2_ is 140 mg g^−1^, and the adsorption process is slow, mainly due to the large particle size of Co(azpy) synthesized by hydrothermal method. It can be seen from Fig. 5(b) that at 295 K and 273 K, the Co(azpy) synthesized by the microwave method almost reaches adsorption equilibrium within 10 min. The equilibrium adsorption capacities at 273 and 295 K are up to 300 and 220 mg g^−1^ within 10 min, respectively. The main factors for the high adsorption capacities of Co(azpy) are attributed to the following aspects. On the one hand, Co(azpy) synthesized by the microwave method possesses small particle size and large specific surface area, which is beneficial to the enhancement of SO_2_ adsorption capacity. On the other hand, the –N=N– group in the Co(azpy) channel, which can be regarded as a Lewis base, has a strong interaction with SO_2_, thereby increasing the adsorption capacity of Co(azpy) to SO_2_. Moreover, the pyridine group in Co(azpy) acts as a basic group and also exhibits a strong interaction with SO_2_, which promotes the improvement of the adsorption capacity of SO_2_. The advantage of the Co(azpy) particles prepared by microwave method compared with synthesis by hydrothermal method is that the adsorption process is fast and less time-consuming.

**Fig. 6:**
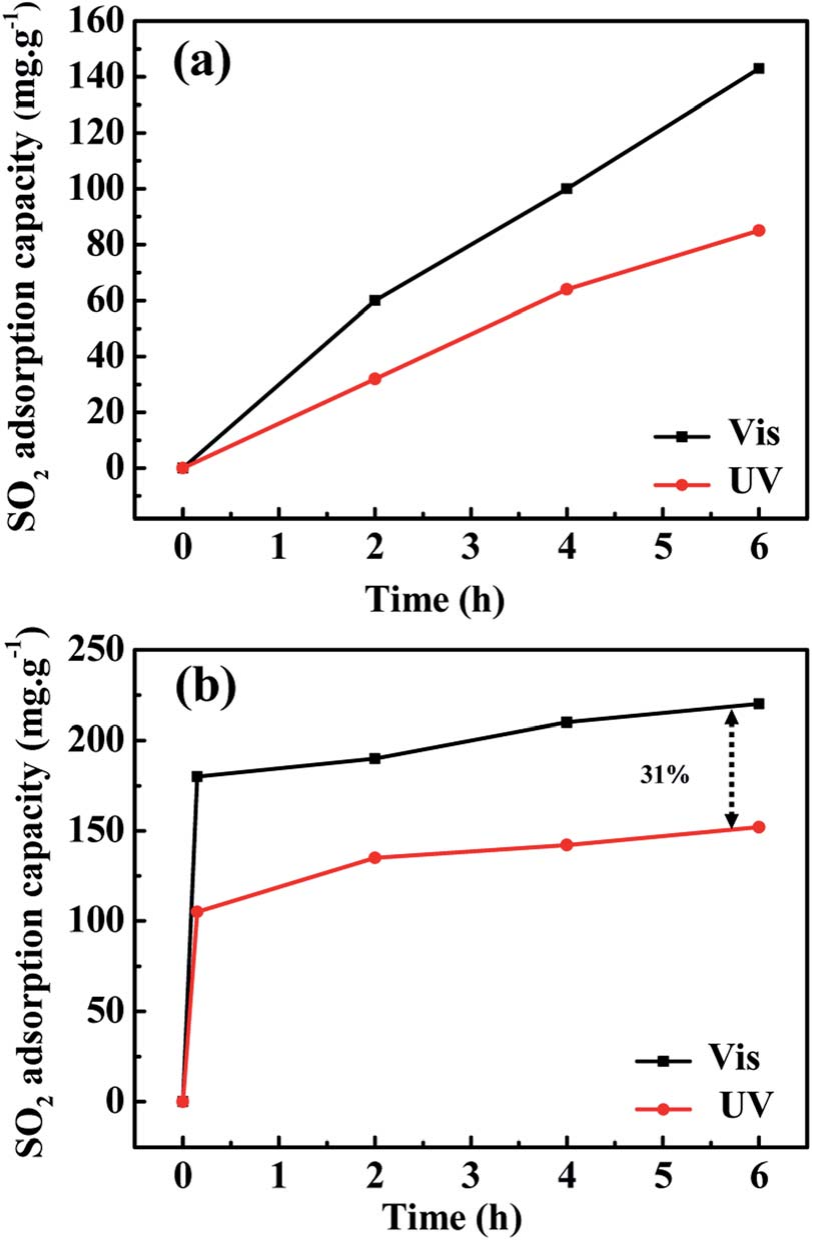
The SO_2_ adsorption capacity of Co(azpy) prepared by (a) hydrothermal method and (b) microwave method under UV-Vis state.

The SO_2_ adsorption capacities of Co(azpy) prepared by hydrothermal method is displayed in Fig. 6(a) at 295 K tested under UV and visible light conditions, respectively. The adsorption capacity under visible light is significantly higher than that under ultraviolet light after 2 h of adsorption test, and the difference in adsorption capacity reaches about 60 mg g^−1^ after 6 h. The SO_2_ adsorption process of Co(azpy) synthesized by microwave method at 295 K is exhibited in Fig. 6(b), and the SO_2_ adsorption capacities are measured both under UV and visible light conditions, respectively. The SO_2_ adsorption capacity of Co(azpy) was increased by ∼31% under visible light irradiation comparison with under ultraviolet light irradiation at 6 h. The increased adsorption of SO_2_ by Co(azpy) under visible light conditions, in comparison with under ultraviolet light, is attributed to the configuration variation. The configuration of Co(azpy) under visible light is *trans*, and it transforms into the *cis* configuration under ultraviolet light. This *trans*–*cis* transition causes variations in the channel structure of Co(azpy) and changes the adsorption capacities of the gas.

### 3.2 Characterization of the membranes

**Fig. 7:**
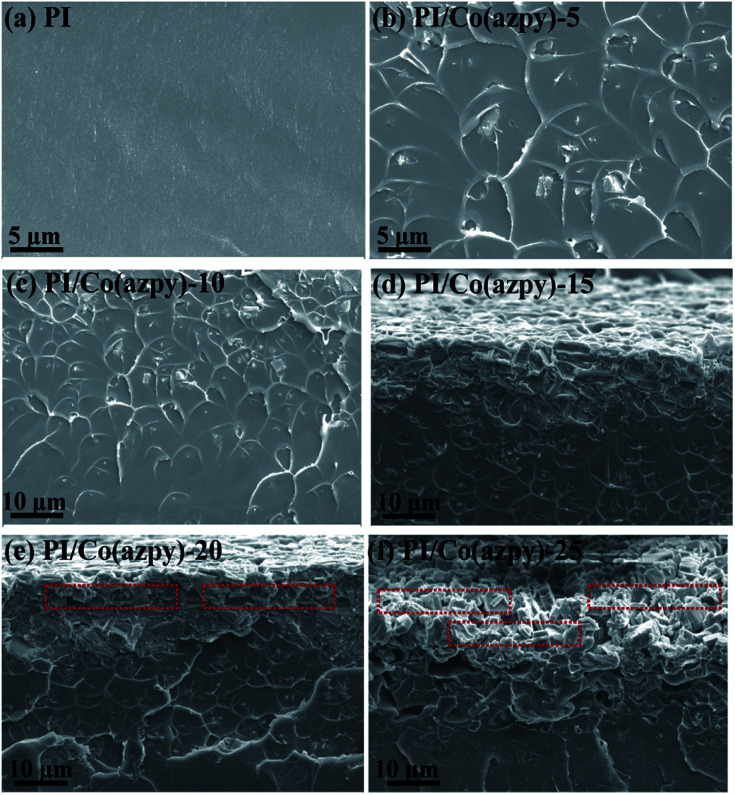
Cross-sectional FESEM images of (a) unfilled PI membrane and MMMs incorporated with (b) 5 wt%, (c) 10 wt%, (d) 15 wt%, (e) 20 wt%, and (f) 25 wt% Co(azpy), respectively.

The cross-sectional morphologies of the unfilled membrane and the MMMs were tested by scanning electron microscope (SEM). The introduction of the light-responsive MOF [Co(azpy)] affects the cross-sectional morphology of the membrane, as shown in Fig. 7. Co(azpy) is gradually deposited in the membrane as the Co(azpy) content increases, causing a large amount of Co(azpy) sheets to accumulate on one side of the membrane. The MMMs with deposition of Co(azpy) still maintain good compactness, and the deposition side of Co(azpy) may accelerate the gas transport in the membrane, including the dissolution and diffusion processes. Generally speaking, the filler begins to coagulate due to the action of gravity as the particle size of the filler is on the micron scale, and the sedimentation process follows Stokes' law.^48^ The particle size of the Co(azpy) sheets filler is 3 μm. Thus, the Co(azpy) sheets in MMMs begin to coagulate as the content exceeds 15%.

**Fig. 8:**
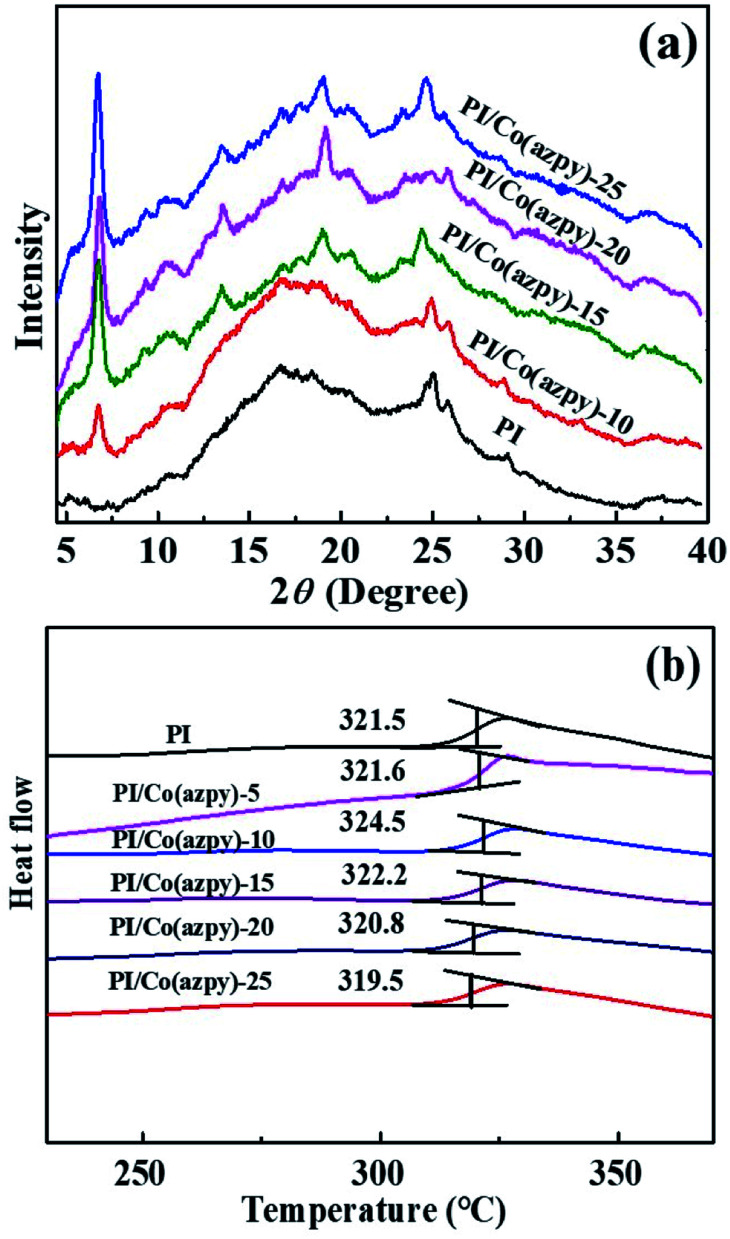
(a) XRD curves and (b) DSC plots of Co(azpy)-incorporated MMMs.

Fig. 8(a) displays the XRD patterns of the unfilled membrane and Co(azpy)-incorporated MMMs, which has a characteristic peak of Co(azpy) at 2*θ* = 7°, and the intensity of the characteristic peak increases with increasing Co(azpy) content. The result indicates that the structure of Co(azpy) remains intact after being doped into the PI matrix. The *T*_g_ of the unfilled PI membrane and MMMs is exhibited in Fig. 8(b). The *T*_g_ of the unfilled PI membrane is about 321.5 °C, and the *T*_g_ of the MMMs is slightly higher than that of the unfilled PI membrane, except for PI/Co(azpy)-25 MMM. This is ascribed to the doping of the Co(azpy) fillers, which causes the PI polymer chain to become rigid to a certain extent, restraining the chain mobility of the polymer. However, the *T*_g_ variations of MMMs are minor, and all within 3°. The highest *T*_g_ of PI/Co(azpy)-10 is 324.5 °C, beneficial to the enhancement of the gas diffusion selectivity, which is attributed to the relatively uniform dispersion of the filler in the membrane.

### 3.3 Gas separation performance

#### 3.3.1 Pure gas separation performance

**Fig. 9:**
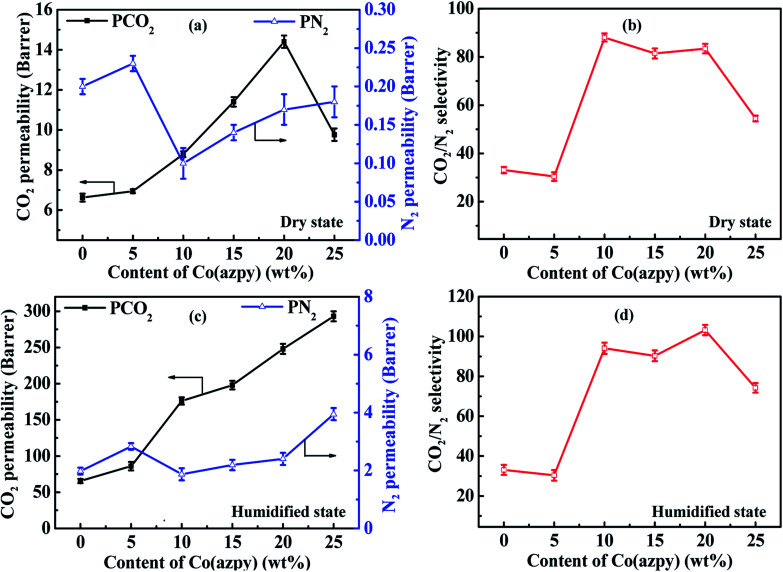
The influence of the Co(azpy) content on the CO_2_/N_2_ separation performance.

The results of the CO_2_/N_2_ separation performance test for the Co(azpy)-filled MMMs both in the dry state and humidified state are shown in Fig. 9. The CO_2_ permeability of the light-responsive MOF [Co(azpy)]-doped MMMs in the dry state increases with the increasing Co(azpy) content, from 6.62 Barrer for the unfilled membrane to 14.4 Barrer for the MMMs loaded with Co(azpy) at a content of 20%. The CO_2_ permeability and CO_2_/N_2_ selectivity of MMMs are 8.79 Barrer and 88, respectively, at the Co(azpy) loading of 10%. The CO_2_ permeability increases to 14.4 Barrer as the Co(azpy) loading ascends to 20%, while the CO_2_/N_2_ selectivity remains basically unchanged in the dry state. The increase in the CO_2_ permeability of MMMs is mainly ascribed to the following factors. Firstly, the presence of pores in Co(azpy) elevates the free volume fraction of the membrane, which is beneficial to the gas diffusion. Secondly, the –N=N– group in the channel of Co(azpy) may promote CO_2_ transport. Thirdly, the pyridine group in Co(azpy) may act as a CO_2_ transport site to accelerate the dissolution and diffusion of CO_2_ in the membrane. Finally, the enhanced CO_2_ permeability is attributed to the junction between Co(azpy) and the PI matrix, which can be used as an extra CO_2_ transport channel. Co(azpy) deposits more obviously in the MMMs as the Co(azpy) content exceeds 20%, resulting in the separation of the two phases, producing non-selective defects, and thus reduces the selectivity to some extent. Before the humidified test, the membranes are pretreated by immersion into water for two weeks. The gas permeabilities of both CO_2_ and N_2_ are significantly enhanced, while the CO_2_/N_2_ selectivity is slightly improved, as shown in Fig. 9(c) and (d), in comparison with MMMs in the dry state. The CO_2_ permeability and CO_2_/N_2_ selectivity reach 293 Barrer and 103.2, respectively. The enhanced gas permeability is ascribed to the enlarged interchain space due to the swollen polymer matrix in the humidified state, which allows for more gas molecules to transport. The slightly enhanced CO_2_/N_2_ selectivity is ascribed to the presence of a basic group from the pyridine ring, which facilitates the CO_2_ transport in the humidified state. The CO_2_/N_2_ selectivity is notably improved, and the remarkably enhanced CO_2_/N_2_ selectivity is attributed to the following aspects. On the one hand, the enhanced CO_2_/N_2_ adsorption selectivity of ∼10 at 1 bar (Fig. 3) is ascribed to the excellent affinity due to the presence of the –N=N– group and pyridine group, which results in the improvement of the CO_2_/N_2_ selectivity. On the other hand, the sheet structure of Co(azpy) provides more gas transport channels for the small molecule CO_2_ rather than the large molecule N_2_, which elevates the CO_2_/N_2_ diffusivity selectivity. Consequently, the interface structure constructed by PI and the Co(azpy) sheet generates more CO_2_ gas transport channels and CO_2_-philic sites, which improves both the CO_2_ permeability and CO_2_/N_2_ selectivity.

#### 3.3.2 Mixed gas separation performance

**Fig. 10:**
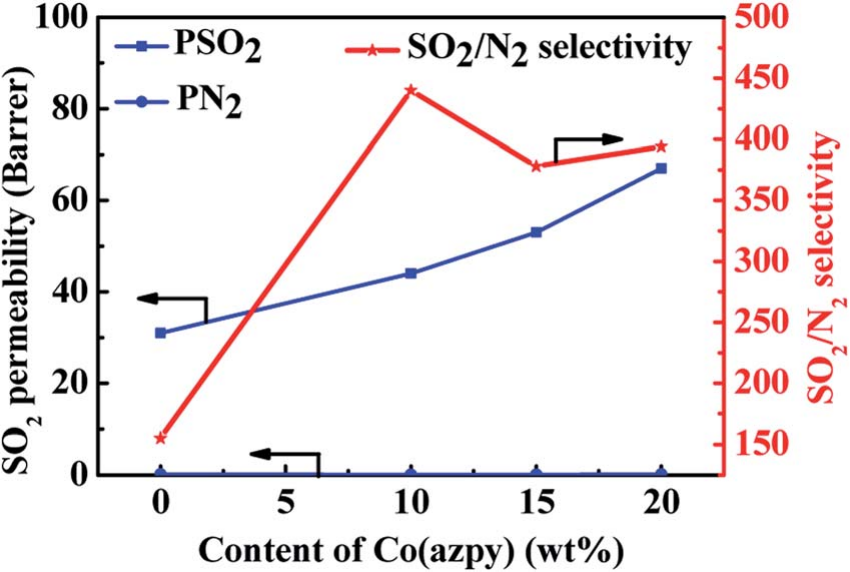
The influence of the Co(azpy) content on the SO_2_/N_2_ separation performance.

The SO_2_/N_2_ separation performance test of the unfilled membrane and MMMs is also shown in Fig. 10. The SO_2_ permeability and SO_2_/N_2_ selectivity of the unfilled membrane and MMMs are higher than that of the CO_2_/N_2_ system. This is because compared with other gases, the permeation process of SO_2_ in the polymer membranes tends to be mainly determined by solubility, not by diffusion rate. The high solubility of SO_2_ is attributed to the high condensability of SO_2_, such as its high critical temperature of 430.8 K, while the critical temperature of CO_2_ is merely 304.2 K. Moreover, the –N=N– group in the channel of the Co(azpy) filler in the MMMs can be regarded as a Lewis base, which has an affinity to SO_2_ and elevates the solubility characteristic of SO_2_. Besides, the pyridine group in Co(azpy) may be used as the facilitated transport site of SO_2_, which accelerates the dissolution and diffusion of SO_2_ in MMMs.

#### 3.3.3 Effect of visible light and ultraviolet light irradiation

**Fig. 11:**
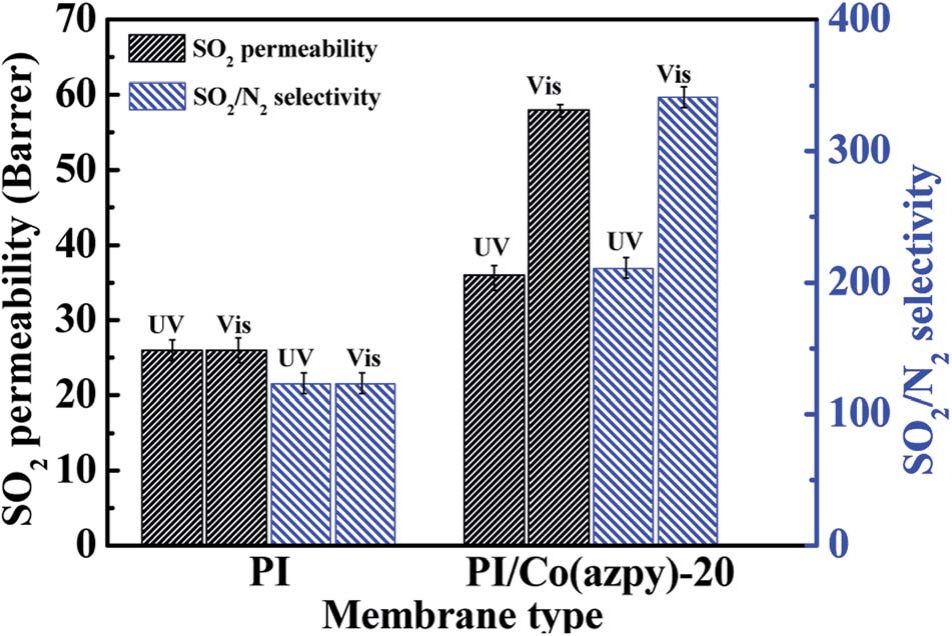
The light-responsive characteristic of the Co(azpy)-loaded MMM for SO_2_/N_2_ separation performance.

**Scheme 4:**
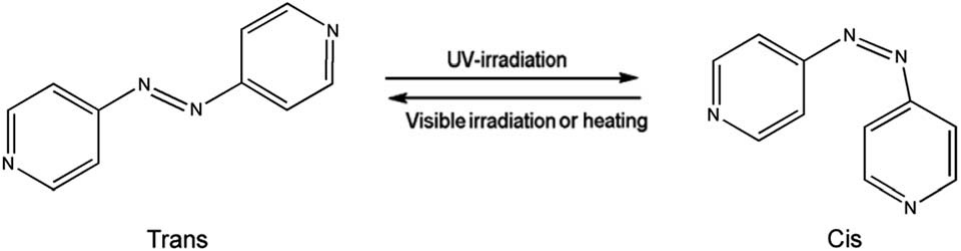
Chemical change of the light-responsive 4,4′-bisazobipyridine (azpy) ligand.

According to the pure gas separation performance test of the membrane, it is concluded that the gas separation performance of the PI/Co(azpy)-20 membrane is the optimum. Thus, the PI/Co(azpy)-20 membrane is used for the photosensitive test. As shown in Fig. 11, under ultraviolet light, the SO_2_ permeability and the SO_2_/N_2_ selectivity of the PI/Co(azpy)-20 membrane is 36 Barrer and 211, respectively. Meanwhile, under visible light, the SO_2_ permeability and the SO_2_/N_2_ selectivity of the PI/Co(azpy)-20 membrane are increased to 58 Barrer and 341, respectively. Under visible light irradiation, the SO_2_ permeability of the PI/Co(azpy)-20 membrane is 61% higher than that under UV light condition, while the change in the N_2_ permeability is basically negligible. This can be attributed to the following aspects due to the incorporation of light-responsive MOF [(Co(azpy) into the MMMs. Co(azpy) may play a role in regulating the size of the channels in MMMs, which is very beneficial to the transport of SO_2_. Co(azpy) possesses a photoactive azo group, which can undergo photo isomerization, as shown in Scheme 4, and change the channel structure of the Co(azpy). Moreover, the –N=N– group and the pyridine group in the channels of Co(azpy) as basic sites can promote the transport of SO_2_. It can be inferred that under ultraviolet light, Co(azpy) is in *cis* configuration and the channel becomes narrow. Meanwhile, under visible light conditions, Co(azpy) is in the *trans* configuration and the channel becomes wide. Consequently, photo isomerization of the light-responsive MOFs simulated by ultraviolet and visible light changes the permeability of the SO_2_ gas in MMMs, which constructs a smart membrane for efficient gas separation.

#### 3.3.4 Comparison of the gas separation performance with the reported literature studies

**Fig. 12:**
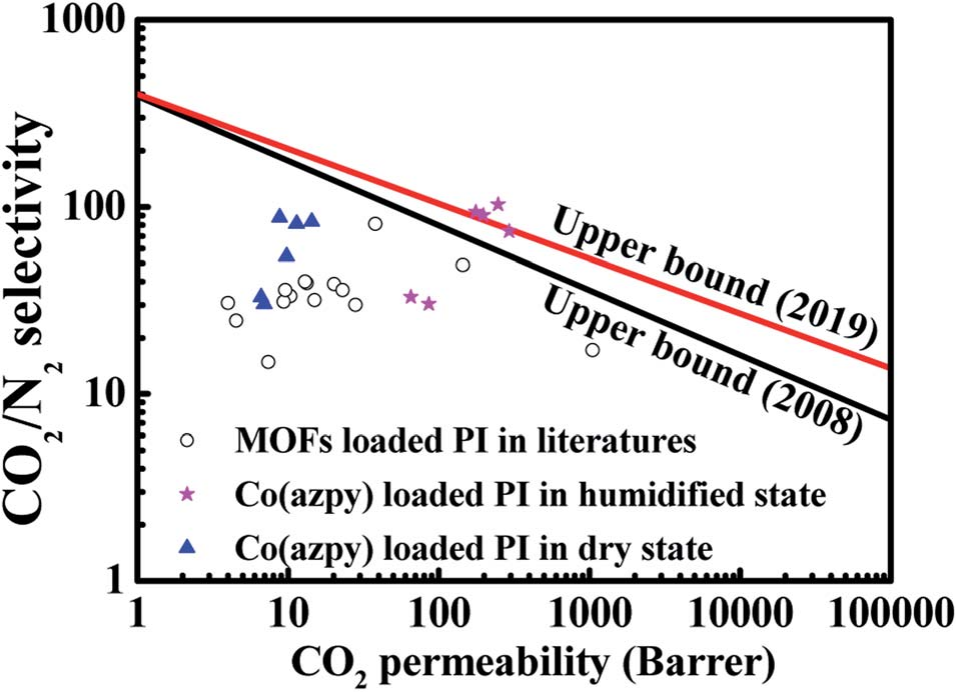
The upper bound of the Co(azpy)-incorporated MMMs for the CO_2_/N_2_ system.

The comparison of the gas separation performance in this study with results reported in the literature is shown in Fig. 12 and Table 1. It has been obviously observed that incorporation of light-responsive MOFs into the Matrimid® 5218 matrix may improve both CO_2_ permeability and the CO_2_/N_2_ selectivity, which may be barely reached by other series of Matrimid® 5218-based MMMs. The CO_2_/N_2_ gas separation performance of light-responsive MOFs sheets-loaded MMMs surpasses the 2019 upper bound redefined by McKeown.

**Table 1:** The Matrimid® 5218-based MMMs for CO_2_/N_2_ separation

Fillers	Loading (wt%)	*T* (°C)	*P* (atm)	*P*_CO_2__ (Barrer)	*α* (CO_2_/N_2_)	Ref
POSS-Zn^2+^	20	35	10	4	30.76	49
C_60_	5	35	10	4.54	24.7	50
Carbon aerogel	30	35	2.6	13.34	39.2	51
Cu-BPY-HFS	30	35	3.9	10.36	33.4	52
MCM-48	10	25	2.5	9.35	31.16	53
MOF-5	30	35	2	20.2	38.8	54
CNTs/GO	5/5	30	2	38.07	81	55
TiO_2_	10	35	2–3	7.4	14.8	56
Azo-DMOF-1	5	35	1.5	9.6	36	57
ZIF-8-DA	40	35	3.5	15	31.8	58
Azo-UiO-66	20	35	4	13	40	40
H-ZIF-8	30	35	2.5	30	—	59
UiO-66	11	—	10	23	36	60
ZIF-68	20	35	10	28	30	61
IPD	20	25	3	1049.4	17.2	62
TiO_2_-PDA-Zn^2+^	7.5	36	6	145	49	63
Co(azpy)	20	30	2	14.4	83.45	This study
				248^a^	103.2^a^	This study

a Humidified membrane.

## 4. Conclusion

In conclusion, one new kind of light-responsive metal–organic frameworks (MOFs)-incorporated smart membrane has been designed and fabricated. The light-responsive metal–organic framework is first synthetized by the microwave method, and the morphology of the sheet can be obtained. The CO_2_ permeability and CO_2_/N_2_ selectivity of the humidified MMMs reach 293 Barrer and 103.2, respectively, which were significantly increased by 3.47-fold and 2.11-fold, respectively, both surpassing the upper bound reported in 2008 and 2019. The light-responsive MOFs sheets are incorporated into commercial polymer materials to fabricate MMMs. In particular, under irradiation with visible light, the azo group in the ligand can be switched from the *cis* to *trans* configuration, contributing to introducing more adsorbed SO_2_ molecules. Moreover, the N_2_ permeability only exhibits slight variation under visible light illumination, while the SO_2_ permeability ascends notably due to the enhanced interaction of the SO_2_ multipole moments with the *trans* light-responsive MOFs, thus resulting in a significant increment of the SO_2_/N_2_ selectivity from 211 to 341. The as-prepared light-responsive MOF-loaded MMM is a promising candidate for highly selective and low energy SO_2_ capture application, which also provides a potential pathway for remote control of the membrane permeability and selectivity by external stimuli.

## Conflicts of interest

There are no conflicts to declare.

## Supplementary Material
